# ‘Blue Carbon’ and Nutrient Stocks of Salt Marshes at a Temperate Coastal Lagoon (Ria de Aveiro, Portugal)

**DOI:** 10.1038/srep41225

**Published:** 2017-01-25

**Authors:** Ana I. Sousa, Danielle B. Santos, Eduardo Ferreira da Silva, Lisa P. Sousa, Daniel F. R. Cleary, Amadeu M. V. M. Soares, Ana I. Lillebø

**Affiliations:** 1 Department of Biology & CESAM – Centre for Environmental and Marine Studies, University of Aveiro, Campus Universitário de Santiago, 3810-193 Aveiro, Portugal; 2 Department of Geosciences & GeoBioTec Research Centre, University of Aveiro, 3810-193 Aveiro, Campus Universitário de Santiago, 3810-193 Aveiro, Portugal; 3 Department of Environment and Planning & CESAM - Centre for Environmental and Marine Studies, University of Aveiro, Campus Universitário de Santiago, 3810-193 Aveiro, Portugal

## Abstract

Ria de Aveiro is a mesotidal coastal lagoon with one of the largest continuous salt marshes in Europe. The objective of this work was to assess C, N and P stocks of *Spartina maritima* (low marsh pioneer halophyte) and *Juncus maritimus* (representative of mid-high marsh halophytes) combined with the contribution of *Halimione portulacoides, Sarcocornia perennis*, and *Bolbochenous maritimus* to the lagoon ≈4400 ha marsh area. A multivariate analysis (PCO), taking into account environmental variables and the annual biomass and nutrient dynamics, showed that there are no clear seasonal or spatial differences within low or mid-high marshes, but clearly separates *J. maritimus* and *S. maritima* marshes. Calculations of C, N and P stocks in the biomass of the five most representative halophytes plus the respective rhizosediment (25 cm depth), and taking into account their relative coverage, represents 252053 Mg C, 38100 Mg N and 7563 Mg P. Over 90% of the stocks are found within mid-high marshes. This work shows the importance of this lagoon’s salt marshes on climate and nutrients regulation, and defines the current condition concerning the ‘blue carbon’ and nutrient stocks, as a basis for prospective future scenarios of salt marsh degradation or loss, namely under SLR context.

‘Blue carbon’ (C) is the coastal C stock, stored in the biomass and sediment of vegetated ecosystems, such as tidal marshes, seagrass beds and mangroves^1^,^2^. These ecosystems play a key role in coastal C sequestration and storage^3^,^4^, contribute to climate regulation at local and global scales^1^,^2^ and to climate change mitigation^5^. Globally, salt marsh sediments have a carbon accumulation rate of ≈245 ± 26 gC m^−2 ^y^−1^ (mean ± SE), while seagrasses have ≈138 ± 38 gC m^−2 ^y^−1^, and terrestrial temperate forests soils ≈5.1 ± 1.0 gC m^−2 ^y^−1^ 6,7,8. Besides providing this important regulating services, salt marshes provide other important ecosystem functions and services, by contributing to shoreline stabilization and protection against erosion, storm surge events and waves or floods^9^. Salt marshes also provide habitats, buffer the effects of potential toxic elements and substances (e.g.^10^) and also act as regulators of nutrient biogeochemical cycles, namely nitrogen (N) and phosphorus (P), contributing to mitigate eutrophication^11^,^12^. However, these habitats are at risk and have been declining due to multiple drivers and pressures. These include natural and anthropogenic disturbances such as extreme storm events, sea level rise (SLR), urbanization, land reclamation for agriculture and eutrophication^13^,^14^. Consequently, all the associated functions and services might be compromised. In addition to this, the sustainability of salt marsh functions and services, including productivity, C storage capacity, nutrient regulation, accretion and sediment elevation gain, may be threatened by SLR in the context of climate change^15^,^16^,^17^.

Ria de Aveiro coastal lagoon is located along the Atlantic Ocean on the northwest coast of Portugal (40°38°N, 8°44°W). It is classified as a Long Term Ecological Research (LTER) site (http://www.lter-europe.net/) and has one of the largest continuous salt marshes in Europe. In this lagoon, *Spartina maritima* (Curt.) Fernald and *Juncus maritimus* Lam. form extensive mono-specific stands, in low- and mid-marshes respectively (Fig. S1A,B from supplementary information). Threats to these habitats have been attributed to eco-hydrological changes due to dredging activity in the main channels of the Ria in order to maintain navigability and the harbour activities. These induced changes in the water current velocity and in tidal prism^18^ have led to increased erosion of the shorelines in some areas of the lagoon and therefore have adversely impacted these habitats (Sousa A.I. & Lillebø A.I. *personal observation*, see Fig. S2). In addition to this, the SLR scenarios for the Ria de Aveiro suggest an increase of 0.42 to 0.64 m in water level by the end of this century, which would represent an increase of 23 to 35% of the lagoon submerged area^19^.

The main objective of this work was to quantify the Ria de Aveiro salt marshes C, N and P stocks, as one of the largest continuous salt marshes in Europe. Its contribution to C storage and nutrient cycles’ regulation, as ecosystem services provided by these coastal areas, was assessed. The specific objectives were: i) assess the C, N and P stocks of *S. maritima* (the low marsh pioneer halophyte) and *J. maritimus* (one of the most representative mid-high marsh halophyte) marshes; and ii) estimate the C, N and P stocks of the whole lagoon’s salt marshes by including the contribution of other mid-high marsh halophyte*s (i.e. Halimione portulacoides* (L.) Aell., *Sarcocornia perennis* (Mill.) A.J. Scott, and *Bolboschoenus maritimus* (L.) Palla). Figure S1 illustrates the halophytes considered in this study. Lastly, threats of SLR in the context of climate change on the salt marshes productivity, C storage capacity and nutrient regulation are briefly discussed.

## Methods

### Study site

**Figure 1:**
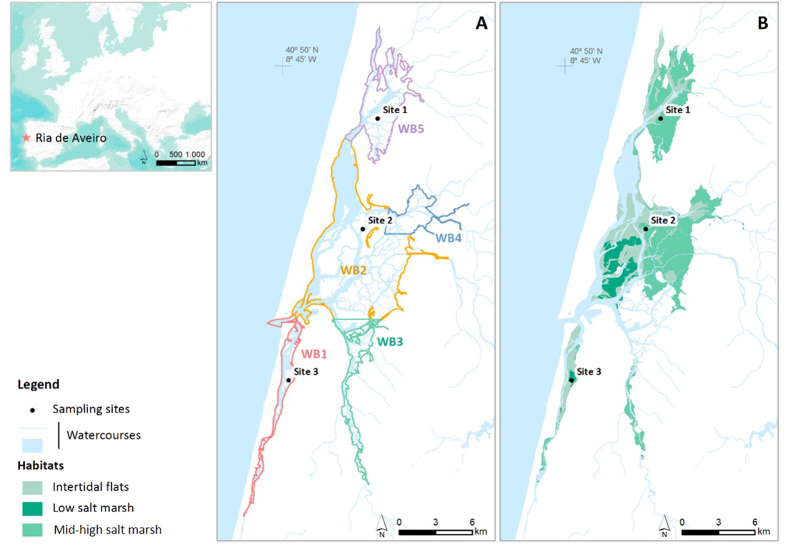
Ria de Aveiro map showing the sampling sites and respective WFD water bodies’ contours (A). Low and mid-high salt marshes distribution at the Ria de Aveiro is shown on the map (**B**). Map generated with ArcGIS 10, (http://www.esri.com/software/arcgis). Credits of World Terrain Base: Esri, USGS, NOAA, DeLorme, NPS.

The Ria de Aveiro is a temperate shallow coastal lagoon (45 km long; 10 km wide) located in a complex coastal region, considering its natural capital, associated socio-economic activities and management^20^,^21^,^22^. Therefore, as a transition area between aquatic and terrestrial ecosystems (ecotone), as well as between seawater and freshwater (ecocline), this coastal lagoon has a large diversity of habitats; complex management issues when defining boundaries and management goals; but also human activities and pressures to deal with^20^,^22^. It is a mesotidal system, with a tidal range from 0.6 m (during neap tides) to 3.2 m (during spring tides) and semi-diurnal tides. The exposed wetland area is about 66 km^2^ at low tide and 83 km^2^ at high tide^18^. The system has a complex geometry characterised by four main channels (Ovar, Espinheiro, Ílhavo and Mira channel) with significant intertidal areas, comprising mud and sand flats, seagrass meadows and salt marshes. Following the EU Water Framework Directive (WFD), the lagoon is divided into five distinct water bodies (WB) (Fig. 1A). This study includes original data from the most representative salt marshes, specifically, site 1 which is located in the northern area of the Ria (Ovar channel, WB5), site 2 which is located in the central area of the Ria (WB2) and site 3 which is located in a southern area (Mira channel, WB1) (Fig. 1B).

### Sampling procedure

*S. maritima* and *J. maritimus* mono-specific stands were sampled at low tide every two months from February to December 2012. At site 1, *S. maritima* was not sampled because the marsh area was too fragmented to be representative (Fig. S2). At each salt marsh, sampling followed the methodology described at Marques *et al*.^10^, i.e., the aboveground biomass (leaves and stems) was collected using 0.3 × 0.3 m^2^ squares (three replicates) and plant litter within this area was also collected. Belowground biomass (roots and rhizomes) was sampled using a steel corer (Ø 8 cm, 25 cm depth) and three corers were collected within each square for a composite sample, minimising the effect of potential spatial variability within each square. All samples were stored separately in plastic bags and transported to the laboratory. Rhizosediment (sediment among roots and rhizomes) temperature and pH were measured *in situ* at each sampling site and date, using a WTW–pH 330i/set equipped with SenTix^®^ 41 (WTW, Weilheim, Germany).

### Laboratorial procedure and stock calculations

Once at the laboratory, belowground biomass was carefully separated from the rhizosediment and both above- and belowground biomass were rinsed with demineralised water. All halophytes biomass was dried at 60 °C until constant weight, ground and homogenised for subsequent analyses. Rhizosediment was air dried, ground and passed through a 0.25 mm mesh. It was subsequently characterized for particle size through sequential sieving, dry bulk density (gDW cm^−3^) and organic matter (OM) content through loss on ignition (LOI%; 6 h combustion at 500 °C)^10^. Total C and N content (live plant biomass and sediment) were quantified in a CHNS/O analyser (Fisons Instruments Model EA 1108, Beverly, Massachusetts, USA), whilst P content was determined following Flindt and Lillebø^23^.

*S. maritima* and *J. maritimus* annual biomass production (for aboveground and belowground), as well as the respective C, N and P annual production was calculated following Sousa *et al*.^11^ and De la Cruz and Hackney^24^. Production refers to the annual increase or decrease in biomass or element stock (C, N or P stock) in a certain area (g m^−2 ^y^−1^). Accordingly, it is calculated by subtracting the minimum stock in a year (mean of all replicates per date), to the maximum stock of the year (mean of all replicates per date). The term stock refers to the biomass or amount of an element (C, N or P) present at a certain time in a certain area (g m^−2^ or Mg ha^−1^) over a particular sediment depth. Particularly, carbon stock is the amount of carbon from organic origin stored in a blue carbon ecosystem^25^. The mean annual C, N and P stocks in the plants were estimated by multiplying C, N, and P contents (%) by the mean annual biomass (g DW m^−2^). Sediment C, N and P stocks (g m^−2^ or Mg ha^−1^) were calculated by multiplying the C, N and P contents (%) per sediment dry bulk density (g DW cm^−3^) and over a particular sediment depth (cm). In turn, annual C, N and P sediment stocks (g m^−2 ^y^−1^ or Mg ha^−1 ^y^−1^) were estimated taking into account the sediment C, N and P content (%), sediment dry bulk density (g DW cm^−3^), but also the sedimentation rate in the salt marsh (cm y^−1^). These data were assessed for the first 25 cm sediment layer and converted to an annual rate (g m^−2 ^y^−1^ or Mg ha^−1 ^y^−1^), thus, integrating the sedimentation process. Therefore, CAR (carbon accumulation rate), as defined in Oyuang and Lee^8^, is comparable to annual C stocks. C, N and P stocks at Ria de Aveiro were also calculated for *Juncus maritimus* and *Spartina maritima* salt marshes (total area), in the plant (aboveground and belowground biomass) and the sediment (first 25 cm layer – it was considered the maximum depth with living belowground biomass - Sousa A.I. & Lillebø A.I., *in situ* observation^11^). *S. maritima* and *J. maritimus* annual average biomass and C, N and P stocks were calculated for the entire lagoon taking into account their relative coverage within each WB. For this purpose all WB were considered, as well as the relative coverage for *J. maritimus* in the mid marsh area (since low marsh is only represented by *S. maritima)*. For the calculation of the relative percentage of halophytes’ floristic composition in each WB, data from Caçador *et al*.^26^ were updated with *in situ* observations. In the Ria WB, transects were done and relative percentage of each halophyte was recorded each meter along transects, following Braun-Blanquet^27^. The three squares of 2.5 m × 2.5 m method per transect was also used as a complementary approach. For the *B. maritimus* annual average biomass and C, N and P stock, data were inferred from a comparable salt marsh area at the Mondego estuary (≈60 Km to the south of the Ria)^11^ taking into account marsh maturity, which corresponds to the best available data comparable to the Ria de Aveiro mid-high marsh. For the calculation of *H. portulacoides* and *S. perennis*, annual average biomass and C, N and P stock data were inferred from a comparable salt marsh area at the Tagus estuary (≈200 Km to the south of the Ria)^11^ taking into account marsh maturity. The mid salt marsh in Tagus is climatologically and environmentally similar to the Ria de Aveiro mid-high marsh, and thus represent the best available data. The salt marsh habitats distribution and mapping by AMBIECO/PLRA – Polis Litoral Ria Aveiro^28^ was updated with *in situ* observations and using ArcGIS 10 (http://www.esri.com/software/arcgis).

### Statistical analyses

In order to assess the halophytes’ annual biomass and nutrient dynamics (including C, N and P cycling and stocks) of *J. maritimus* and *S. maritima*, a table containing the following variables (‘Temp’ (sediment temperature), ‘LOI’, ‘pH’, ‘G1000’, ‘G500’, ‘G250’, ‘G125’, ‘G0063’ (sediment particle size: >1 mm, >500 μm to ≤1 mm, >250 to ≤500 μm, >125 to ≤250 μm, >63 to ≤125 μm, and ≤63 μm, respectively) ‘AG_B’, ‘BG_B’, ‘D_B’ (above, belowground and litter biomass, respectively), ‘AG_C’, ‘BG_C’ (above and belowground C content, respectively), ‘AG_N’, ‘BG_N’ (above and belowground N content), ‘AG_P’, ‘BG_P’ (above and belowground P content), ‘S_C’, ‘S_N’ and ‘S_P’ (sediment C, N and P stock in the first cm depth, respectively)), was imported into R using the read.table() function. Data variability was accounted on the analysis by including all replicate data for each measured variable (N = 3). This matrix was log_e_ (*x* + 1) transformed (in order to normalise the distribution of the data) and a distance matrix was constructed using the Euclidean index with the vegdist() function in the VEGAN package^29^ in R. Variation in variable composition among sampling events and sites was assessed with Principal Coordinates Analysis (PCO) using the cmdscale() function in R with the Euclidean distance matrix as input. Separate ordinations were generated for *J. maritimus* only (*Juncus*), *S. maritima* only (*Spartina*) and both species. For *J. maritimus* only and *S. maritima* only, we tested for significant variation (α = 0.05) among sampling events using the adonis() function in VEGAN. In the adonis analysis, the Euclidean distance matrix was the response variable with the variable ‘sampling event’ as independent variable. The number of permutations was set at 999; all other arguments used the default values set in the function. For both species together, we tested for significant variation in variable composition among sampling events and between both species (*J. maritimus* and *S. maritima*) using the sites where both species were present (sites 2 and 3). Weighted averages scores were computed for environmental variables on the first two PCO axes using the wascores() function in the vegan package. Detailed descriptions of the functions used here can be found in R (e.g., ?cmdscale) and online in reference manuals (http://cran.r-project.org/web/packages/vegan/index.html; 2015/05/29).

## Results

### Salt marshes characterization

**Figure 2:**
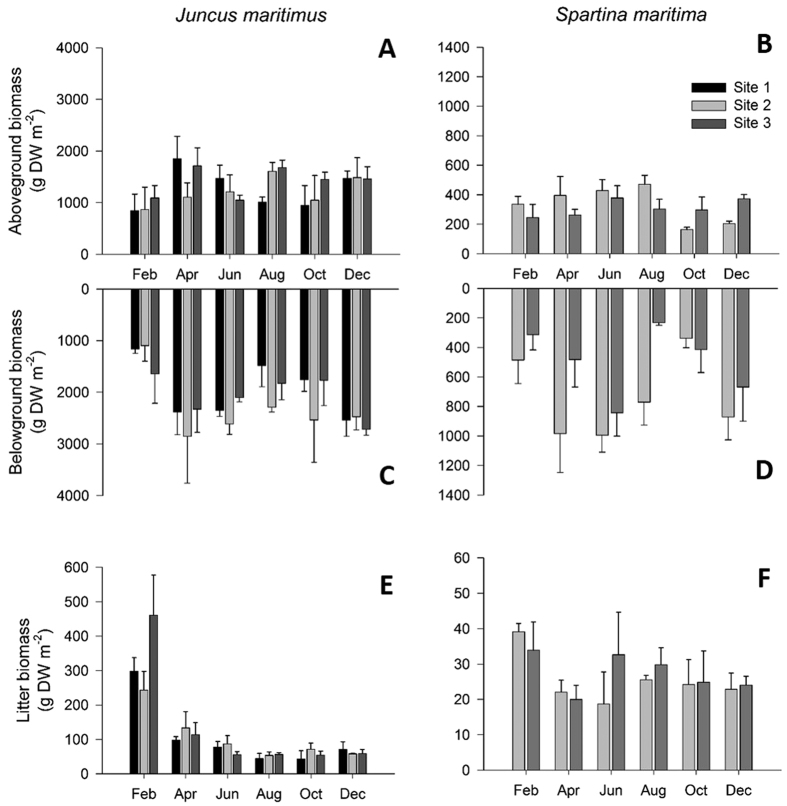
*Juncus maritimus* (A,C,E) and *Spartima maritima* (B,D,F) aboveground biomass (leaves and stems; A,B), belowground biomass (roots and rhizomes; C,D) and biomass of leaf litter remaining in the marsh (E,F), along sampling period at sites 1, 2 and 3. Please note different scales in graphs. Mean values (N = 3) and standard error (SE) are shown.

**Figure 3:**
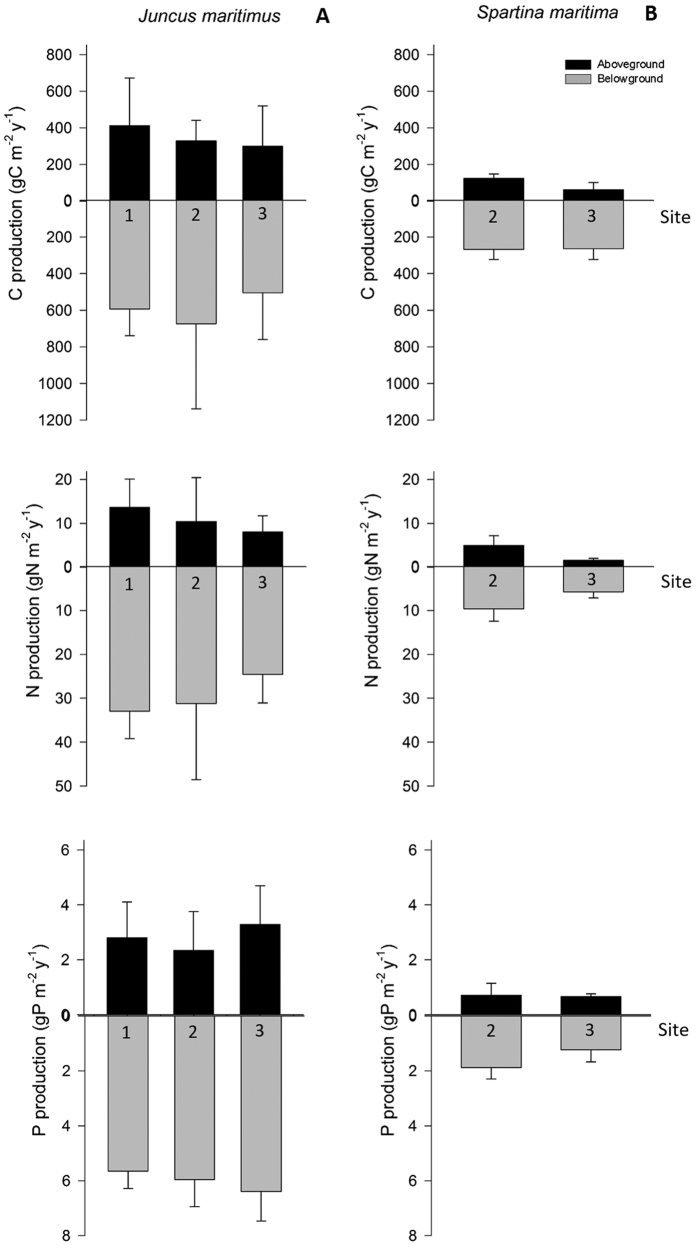
C, N and P production by *Juncus maritimus* (A) and *Spartina maritima* (B) at sites 1, 2 and 3 of the Ria de Aveiro lagoon. Aboveground includes the halophyte stems and leaves, and belowground includes roots and rhizomes. Mean values (N = 18) and standard error (SE) are shown.

The mean sediment temperature was lowest in December (8.5 ± 1.6 °C) and highest in June (22.2 ± 3.2 °C) in both sites. The pH at *S. maritima* and *J. maritimus* rhizosediment ranged between 6.1 ± 0.1–7.2 ± 0.0 and 5.9 ± 0.0–7.0 ± 0.2 (mean ± standard deviation (SD)), respectively. The organic matter (%) in *S. maritima* and *J. maritimus* rhizosediment ranged between 5.9 ± 1.0%–12.3 ± 1.6% and 11.9 ± 1.2%–28.2 ± 2.4%, mean ± SD), respectively. In low marshes (*S. maritima*), sediment particle size ranged from 36% to 61% silt and clay, whereas in *J. maritimus* marshes mean silt and clay content ranged from 52% to 57%. The *in situ* environmental parameters and sediment characterization are shown in detail in Table S1. Belowground biomass was generally higher than the aboveground biomass for both halophytes, with *J. maritimus* having higher biomass, as well as litter biomass (Fig. 2). In the lagoon, maximum total biomass for *J. maritimus* was recorded between April and August, whilst for *S. maritima* it was between April and June. Both species showed a slight increase in biomass (but lower than maximum) in December and the highest litter biomass in February. Following plant annual biomass, the net annual C, N and P production of both halophytes was higher in the belowground and higher in *J. maritimus* (Fig. 3A,B).

**Figure 4:**
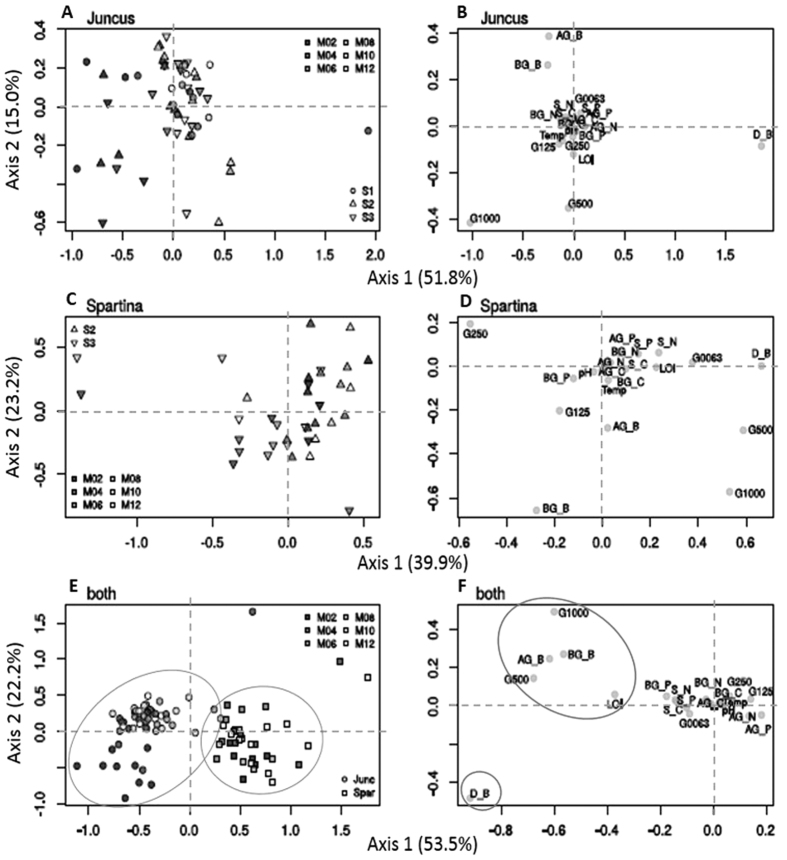
Principal Coordinates Analysis (PCO) showing the variation among tested environmental variables: sediment temperature (‘Temp’); organic matter/loss on ignition (‘LOI’); pH; sediment particle size: >1 mm, >500 μm to ≤1 mm, >250 to ≤500 μm, >125 to ≤250 μm, >63 to ≤125 μm, and ≤63 μm (‘G1000’, ‘G500’, ‘G250’, ‘G125’, ‘G0063’, respectively); above, belowground and litter biomass (‘AG_B’, ‘BG_B’, ‘D_B’, respectively); above and belowground C content (‘AG_C’, ‘BG_C’, respectively); above and belowground N content (‘AG_N’, ‘BG_N’, respectively); above and belowground P content (‘AG_P’, ‘BG_P’, respectively) and sediment C, N and P stock in the first cm depth (‘S_C’, ‘S_N’ and ‘S_P’, respectively). Separate ordinations generated for *J. maritimus* only (**A**,**B**), *S. maritima* only (**C**,**D**) and for both species (**E**,**F**) are shown.

With respect to the annual biomass and nutrient dynamics of *J. maritimus*, the PCO (Fig. 4A,B), showed that there are no clear differences among sampling sites and sampling dates. Some observed variation appears related to litter biomass and the coarser sediment fraction G1000 (1000 μm > grain size > 500 μm) (axis 1 ≈ 52%). The annual biomass and nutrient dynamics of *S. maritima* differs somewhat among sampling sites, although this difference among sites is not clear. The observed variation appears related to litter and the sediment fraction G250, G500 and G1000 (grain size > 125 μm) (axis 1 ≈ 40%). Seasonal differences are, however, not apparent (Fig. 4C,D). The PCO analysis clearly separates *J. maritimus* and *S. maritima* (Fig. 4E,F). This separation is related to differences in plant aboveground, belowground and litter biomass, sediment granulometry (G500 and G1000) and organic matter content (LOI). Again, differences among sampling dates were not apparent. Overall, for each halophyte, there was no apparent relationship between its annual biomass and nutrient dynamics, C, N and P stocks, and sampling dates.

### Salt marshes ‘blue carbon’ and nutrient stocks

**Figure 5:**
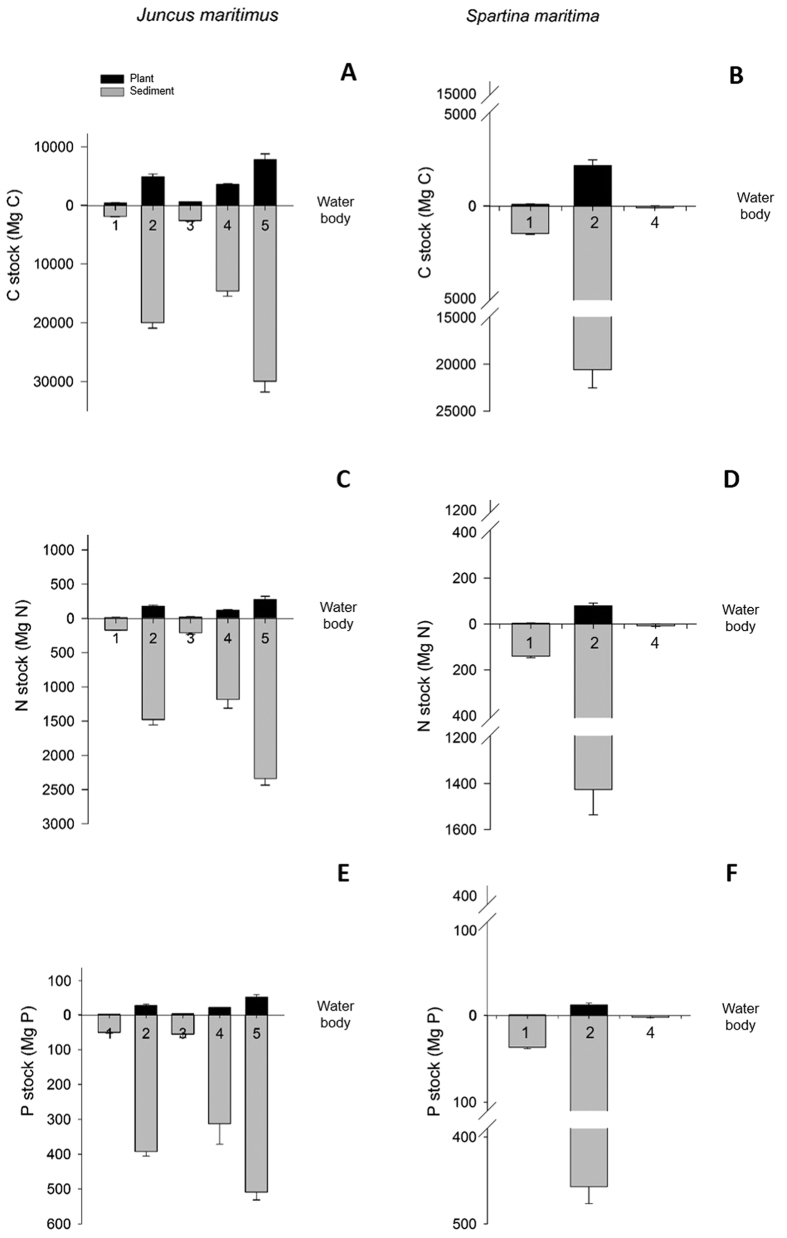
Total C, N and P stock at Ria de Aveiro *Juncus maritimus* and *Spartina maritima* salt marshes, in the plant (aboveground and belowground biomass) and the sediment (0–25 cm depth), per water body (WB) total area. Mean values (N = 18) and standard error (SE) are shown.

‘Blue carbon’ and nutrient (N and P) dynamics differed between species but were similar among the studied sites. We, therefore, extrapolated stock values to marshes in the whole lagoon, taking into account the relative coverage of each halophyte. Mean annual C, N and P stocks in *J. maritimus* and *S. maritima* biomass in marshes per water body (WB) are shown in Fig. S3 from Supplementary information. Figure 5 illustrates C, N and P stocks in the biomass of the halophytes and in the respective rhizosediment. *J. maritimus* C and nutrient stocks are much higher than those in *S. maritima* marshes, with *J. maritimus* storing on average seven to eight times higher more ‘blue carbon’ and nutrients (N and P) in the biomass, and three times higher in the rhizosediment (Fig. 5).

In addition to ‘blue carbon’ and nutrient stock assessment for *J. maritimus* and *S. maritima*, mean stocks for *H. portulacoides, S. perennis* and *B. maritimus* were also estimated (Table S2). The total mean stock (plant and sediment) was highest for C in *J. maritimus* (1517 ± 26 gC m^−2 ^y^−1^ mean ± SE) and for N and P in *H. portulacoides* (109 gN m^−2 ^y^−1^ and 29 gP m^−2 ^y^−1^). *S. maritima* had the lowest C, N and P stocks (493 ± 20 gC m^−2 ^y^−1^, 23.3 ± 0.1 gN m^−2 ^y^−1^ and 5.0 ± 0.1 gP m^−2 ^y^−1^ mean ± SE).

Considering the whole lagoon salt marsh area (≈4400 ha) the total mean C, N and P stocks, including the five most representative plant species, are: 252053 Mg C; 38100 Mg N and 7563 Mg P (Table 1). Overall, more than 90% of C, N and P are stocked in the mid-high marsh (93–97% of the plant stocks and 90–96% of the rhizosediment stocks).

## Discussion

Salt marsh halophytes can alter rhizosphere pH through oxygen transfer to the roots and rhizomes and thus, lead to temporal and spatial changes in rhizosediment chemistry (e.g. ref. 30). This is related to halophytes-specific processes^31^ and related to their seasonal changes in biomass and nutrient dynamics. For instance, *S. maritima* as a pioneer species is able to colonize bare mudflat anoxic sediments, which have higher pH, and subsequently, lead to the rhizosediment oxidation through oxygen transport to the roots and rhizomes^32^. The annual biomass and nutrient dynamics of *J. maritimus* and *S. maritima* show that *J. maritimus* had higher biomass (above- and belowground), which in turn was related to higher C, N and P stocks, both in the plant and rhizosediment. High belowground biomass in *J. maritimus* marshes is also a source of detritus, leading to a higher organic matter content in the sediment^32^,^33^.

In the Ria the belowground biomass of *S. maritima* and *J. maritimus* marshes was always higher than the respective aboveground biomass. The *S. maritima* average belowground/aboveground biomass ratio is 1.9, which is comparable to the Mondego estuary Jusante marsh (2.1), slightly lower than the Tagus estuary Pancas marsh (2.7) and much higher than the Mondego estuary Gala marsh (0.4)^32^. This ratio at the Ria is much lower than at the Odiel *S. maritima* natural marshes in Huelva, Spain (6.3 and 9.7)^34^, and than at Tagus estuary Corroios marsh (19)^32^. These differences may be due to marsh elevation, related to marsh maturity^35^, to the hydrodynamics of the system (e.g. hydrology, flooding frequency and duration) and to the chemical environment (e.g. nutrient availability, redox potential, toxic elements)^11^,^32^,^36^. *J. maritimus* aboveground/belowground biomass ratio in Ria is 1.6. To our best knowledge, there are no works with comparable sampling methodology, disabling possible comparisons.

Besides the considered environmental variables, comparable sampling methods is of paramount importance^37^ and comparisons of biomass, ‘blue carbon’ and nutrient stocks among marshes must take this into account. Accordingly, our results are discussed with others wherein the same or similar methods were applied, or if considerably different, this is discussed.

There was no clear spatial and temporal variation within *S. maritima* and *J. maritimus* marshes. Differences, however were clear between halophytes and mainly due to higher biomass and higher organic matter in *J. maritimus* sediment. *S. maritima* low marshes occur in areas protected from high-energy wave action, between mean sea level and mean high tide; *J. maritimus* mid-high marshes occur from mean high water level until the upper reaches of spring tides. C, N and P dynamics and stocks differ in both marshes, with overall (plant and rhizosediment) C, N and P stocks higher in *J. maritimus* (less influenced by tidal action dynamics, flooded twice a day but only at spring tides) than in *S. maritima* marshes (flooded twice a day at neap and spring tides following the tidal cycle). In addition to this, marshes constitute a barrier for the settling of upstream freshwater suspended particles that is a supply of organic rich matter. Both processes combined might also contribute to comparatively higher ‘blue carbon’ and nutrient stocks in mid-high marshes. The C accumulation rate was shown to depend on three main drivers: sediment (fine particles) accumulation rate, dry bulk density and sediment organic C content^8^.

The annual C stock (also known as CAR, C accumulation rate) in *S. maritima* rhizosediment (120 gCm^−2^y^−1^) is lower than values recorded at other temperate marshes, such as the Mondego (218 gCm^−2^y^−1^) and Tagus estuaries (330 and 750 gCm^−2^y^−1^), in Portugal^38^ and at Huelva Estuary restored marshes (273 gCm^−2^y^−1^)^39^, in Spain. Also, the *J. maritimus* C stock in the sediment is lower (133 gCm^−2^y^−1^) than recorded at Riverine sites along the Rhone Delta (France) (357 gCm^−2^y^−1^)^40^. Differences may be due to geophysical processes and other environmental variables as discussed in the meantime. Sediment deposition and plant productivity contribute to vertical marsh accretion^41^, increasing the C stock in the sediment. Actually, carbon accumulation in the sediment has been shown to increase with belowground biomass production and, on contrary, decrease with organic matter decomposition^6^,^38^,^42^. Therefore, final C stock in the salt marsh sediment depends on the balance between these processes. Decomposition of organic matter in the sediment was recorded to increase with increasing temperatures, as a result of climate change, thus decreasing the sediment C stock^43^,^44^. Nevertheless, Kirwan *et al*.^45^ suggested that, at higher latitudes, higher temperatures may increase the halophytes’ productivity, thus increasing the sediment C stock and counteracting the loss of C as a result of decomposition.

*S. maritima* at a Tagus salt marsh (Corroios, Portugal) showed a low detritus decomposition rate (k = 0.0024, after 180 days) and a high belowground biomass production which, combined with specific physical-chemical parameters, led to a high organic matter accumulation and high C stock in the sediment, when compared to other Portuguese salt marshes^38^. However, depending on the mentioned parameters, decomposition rates may be faster still for *S. maritima* detritus (e.g. k = 0.0043, at a Mondego estuary salt marsh (Portugal))^38^, but also for *J. maritimus*, whose decomposition rate was shown to be 0.033 at a Ria de Aveiro salt marsh^10^. In these works, decomposition rates were assessed for the more labile C fraction. Although it is not possible to estimate the relative contribution of low marshes, the Ria contributes to a net export of C associated with plant detritus to the Atlantic Ocean^46^.

In addition to this, the estimated annual net export of organic C in the water column, in the particulate (POC) and dissolved forms (DOC) is approximately 7957 tonnes y^−1^ (6585 tonnes y^−1^ POC + 1372 tonnes y^−1^ DOC); this corresponds to approximate annual net flux of 79 g m^−2^ of POC and 17 g m^−2^ of DOC^47^.

N stock in *S. maritima* sediment (9.9 ± 2.2 gNm^−2^y^−1^) is lower in the Ria than in the Mondego estuary (23 to 25 gNm^−2^y^−1^), the Tagus estuary (29 to 61 gNm^−2^y^−1^)^32^ and the restored marshes in Huelva estuary (36.2 gNm^−2^y^−1^)^39^. N stock in *J. maritimus* rhizosediment in the Ria is 10.8 ± 1.4 gm^−2^y^−1^. To our best knowledge there is no comparable data in the literature for *J. maritimus* N stocks. P stocks in *S. maritima* rhizosediment in the Ria (2.8 ± 0.37 gPm^−2^y^−1^) were lower than Mondego (6 gPm^−2^y^−1^) and Tagus estuaries (9 gPm^−2^y^−1^)^11^. *J. maritimus* rhizosediment P stock in the Ria is 2.8 ± 0.7 gPm^−2^y^−1^. Also, no comparable data for *J. maritimus* P stocks was found in the literature. Differences in *S. maritima* N and P stocks may be due to site-specific hydrological, geomorphological characteristics and geophysical processes, all relevant to marsh ecological processes, as shown by^11^.

Considering the Ria de Aveiro as a whole, differences in the stocks among halophyte species are due to higher biomass, higher relative coverage within the marsh and the species ubiquity at Ria de Aveiro marshes. *S. maritima* forms clear monospecific stands at three WB, while *J. maritimus* forms clear monospecific stands at the five WB. In some WB, *J. maritimus* co-occur with *H. portulacoides, B. maritimus* and *S. perenis*. Total ‘blue carbon’ and nutrient stocks estimated for the Ria de Aveiro salt marshes total area are 252053 Mg C, 38100 Mg N and 7563 Mg P. From these, more than 90% is stored at mid-high marshes.

The estimations of the total ‘blue carbon’ and nutrient stocks for the whole Ria de Aveiro salt marshes demonstrate the contribution of these marshes to climate and nutrient regulation, as crucial regulation and maintenance ecosystem services, but also allow to define the current condition of these marshes for prospective future scenarios, namely under SLR context. As previously mentioned, shorelines erosion in certain areas of the lagoon are already occurring (see Fig. S2), meaning that part of these C and nutrient stocks in the salt marshes may be adversely impacted, being at risk. In fact, the ability of salt marshes to cope with SLR and their future sustainability depends on many dynamic and interacting processes influencing the potential for wetland submergence (e.g. vertical accretion, ability to migrate inland)^7^,^44^,^48^,^49^. Therefore, in addition to the expected SLR, other variables must be considered when assessing salt marsh vulnerability and resilience. Namely, sediment accumulation, plant growth and biomass, and decomposition rate affect vertical accretion^7^,^49^ and the wetland surface elevation in salt marshes. Many models for coastal systems, including a hydrodynamic model for the Ria de Aveiro^50^,^51^, have focused on the predicted SLR, highlighting these systems vulnerability (e.g.^52^,^53^), however not considering salt marsh vulnerability and resilience. According to Kirwan and Mudd^44^, coastal C burial rates are expected to increase in the first half of the 21^st^ century, as a result of climate change (including SLR), while in a long-term perspective, this feedback is predicted to decrease over time, thus decreasing C burial rates. Kirwan *et al*.^54^ also highlighted recently the importance of considering different biophysical feedback processes occurring in salt marshes for SLR scenarios. However, not all studies consider the associated ecosystem ecological response. We, therefore, reinforce the need to consider human management actions (past and future) that may have synergistic effects with climate change (SLR) *versus* the saltmarshes vulnerability and resilience, with consequent implications in ‘blue carbon’ and nutrient stocks.

## Conclusions

At Ria de Aveiro coastal lagoon salt marshes, the C, N and P stocks of *S. maritima* are lower than *J. maritimus*. Total ‘blue carbon’, N and P stocks estimated for all Ria marshes (considering the five most representative halophytes) are 252053 Mg, 38100 Mg and 7563 Mg, respectively, with mid-high marshes corresponding to 90% of the total stocks. This study defines the current condition concerning the ‘blue carbon’ and nutrient stocks at Ria de Aveiro lagoon salt marshes, highlighting its contribution to climate and N and P cycles regulation, but is also a basis for prospective future scenarios of salt marsh degradation, namely under SLR context. The need to consider multiple stressors (natural or anthropogenic related) at different temporal and spatial scales combined with biophysical feedback processes, when considering potential impacts on the salt marshes ‘blue carbon’ and N and P stocks is also reinforced.

## Additional Information

**How to cite this article**: Sousa, A. I. *et al*. ‘Blue Carbon’ and Nutrient Stocks of Salt Marshes at a Temperate Coastal Lagoon (Ria de Aveiro, Portugal). *Sci. Rep.* **7**, 41225; doi: 10.1038/srep41225 (2017).

**Publisher's note:** Springer Nature remains neutral with regard to jurisdictional claims in published maps and institutional affiliations.

## Supplementary Material

Supplementary Information

## Figures and Tables

**Table 1:** Total C, N and P stocks (mean in Mg) in the plant (above- and belowground biomass) and sediment (0–25 cm depth), for the all Ria de Aveiro salt marshes area.

	Stock compartment	Low marsh	Mid/high marsh				Ria de Aveiro marshes
		*Spartina maritima*	*Juncus maritimus*	*Halimione portulacoides*	*Sarcocornia perennis*	*Bolboschoenus maritimus*	
C	Plant	2326	17368	13118	1946	948	35706
	Sediment	22188	69017	104102	14578	6461	216346
	**TOTAL**	**24514**	**86385**	**117220**	**16524**	**7409**	**252052**
			**227538**				
N	Plant	84	619	678	78	30	1489
	Sediment	1576	5385	25442	3590	619	36312
	**TOTAL**	**1660**	**6004**	**26120**	**3668**	**649**	**38101**
			**36441**				
P	Plant	13	110	349	33	5	510
	Sediment	497	1320	4559	516	160	7052
	**TOTAL**	**510**	**1430**	**4908**	**549**	**165**	**7562**
			**7052**				

Mean values are shown (N = 18 for *Juncus maritimus* and *Spartina maritima*; N = 15 for *Halimione portulacoides, Sarcocornia perennis* and *Bolboschoenus maritimus*).

## References

[b1] Nellemann C., Corcoran E., Duarte C. M., Valdés L., De Young C., Fonseca L. & Grimsditch G. Blue Carbon: the Role of Healthy Oceans in Binding Carbon. A Rapid Response Assessment. United Nations Environment Program (2009).

[b2] Pendleton L. . Estimating Global “Blue Carbon” Emissions from Conversion and Degradation of Vegetated Coastal Ecosystems. PLoS One 7(9), e43542 (2012). 22962585 10.1371/journal.pone.0043542 PMC3433453

[b3] Herr D., Pidgeon E. & Laffoley D. Blue Carbon Policy Framework: Based on the discussion of the International Blue Carbon Policy Working Group Gland, Switzerland, IUCN and Arlington, USA: CI 39pp. (2011)

[b4] Beaumont N. J., Jones L., Garbutt A., Hansom J. D. & Toberman M. The value of carbon sequestration and storage in coastal habitats. Estuar Coast Shelf S 137, 32–40 (2014).

[b5] Duarte C., Losada I. J., Hendriks I. E., Mazarrasa I. & Marbà N. The role of coastal plant communities for climate change mitigation and adaptation. Nat Clim Change 3, 961–968 (2013).

[b6] Mcleod E. . A blueprint for blue carbon: toward an improved understanding of the role of vegetated coastal habitats in sequestering CO2. Front Ecol Environ 9(10), 552–560 (2011).

[b7] Chmura G. L. What do we need to assess the sustainability of the tidal salt marsh carbon sink? Ocean Coast Manage 83, 25–31 (2013).

[b8] Ouyang X. & Lee S. Y. Updated estimates of carbon accumulation rates in coastal marsh sediments. Biogeosciences 11, 5057–5071 (2014).

[b9] Shepard C. C., Crain C. M. & Beck M. W. The Protective Role of Coastal Marshes: A Systematic Review and Meta-analysis. PLoS One 6(11), e27374 (2011). 22132099 10.1371/journal.pone.0027374 PMC3223169

[b10] Marques B., Lillebø A. I., Pereira E. & Duarte A. C. Mercury cycling and sequestration in salt marshes sediments: an ecosystem service provided by Juncus maritimus and Scirpus maritimus. Environ Pollut 159, 1869–1876 (2011). 21514707 10.1016/j.envpol.2011.03.036

[b11] Sousa A. I., Lillebø A. I., Pardal M. & Caçador I. Productivity and nutrient cycling in salt marshes: contribution to ecosystem health. Estuar Coast Shelf S 87, 640–646 (2010).

[b12] Shao X., Wu Ming, Binhe Gu, Yinxu Chen & Xinqiang Liang Nutrient retention in plant biomass and sediments from the salt marsh in Hangzhou Bay estuary, China. Environ Sci Pollut R 20, 6382–6391 (2013). 10.1007/s11356-013-1698-6 23589271

[b13] Deegan L. A. . Coastal eutrophication as a driver of salt marsh loss. Nature 18, 490(7420), 388-92 (2012). 10.1038/nature11533 23075989

[b14] Nelson J. L. & Zavaleta E. S. Salt Marsh as a Coastal Filter for the Oceans: Changes in Function with Experimental Increases in Nitrogen Loading and Sea-Level Rise. PLoS One 7(8), e38558 (2012). 22879873 10.1371/journal.pone.0038558 PMC3413704

[b15] Kirwan M. L. & Megonigal J. P. Tidal wetland stability in the face of human impacts and sea-level rise. Nature 504, 53–60 (2013). 24305148 10.1038/nature12856

[b16] Langley J. A., Mozdzer T. J., Shepard K. A., Hagerty S. B. & Megonigal J. P. Tidal marsh plant responses to elevated CO2, nitrogen fertilization, and sea level rise Glob. Change Biol. 19, 1495–503 (2013). 10.1111/gcb.12147 23504873

[b17] Caplan J. S., Hager R. N., Megonigal J. P. & Mozdzer T. J. Global change accelerates carbon assimilation by a wetland ecosystem engineer. Environ. Res. Lett., 10, 115006 (2015).

[b18] Picado A., Dias J. M. & Fortunato A. B. Tidal Changes in estuarine systems induced by local geomorphologic modifications. Cont Shelf Res 30(17), 1854–1864 (2010).

[b19] Lopes C. L., Azevedo A. & Dias J. M. Flooding assessment under sea level rise scenarios: Ria de Aveiro case study. J Coastal Res SI65, 766–771 (2013).

[b20] Lillebo A. I. . In Coastal Lagoons in Europe: Integrated Water Resource Strategies (eds Lillebo A. I., Stalnacke P. & Gooch G. D.) Ch. 3, 21–29 (IWI Publishing, 2015).

[b21] Dolbeth M. . An integrated Pan-European perspective on coastal Lagoons management through a mosaic-DPSIR approach. Sci. Rep. 6, 19400, doi: 10.1038/srep19400 (2016). 26776151 PMC4725967

[b22] Sousa L. P. . Ecosystem services provided by a complex coastal region: challenges of classification and mapping. Sci. Rep. 6, 22782, doi: 10.1038/srep22782 (2016). 26964892 PMC4786800

[b23] Flindt M. R. & Lillebø A. I. In Methods to Study Litter Decomposition (eds Graça M. A. S., Bärlocher F. G. & Mark O.) (Dordrecht, Springer, 2005).

[b24] De la Cruz A. A. & Hackney C. T. Energy value, elemental composition, and productivity of belowground biomass of a Juncus tidal marsh. Ecology 58, 1165–1170 (1977).

[b25] Howard J., Hoyt S., Isensee K., Telszewski M. & Pidgeon E. Coastal Blue Carbon: Methods for assessing carbon stocks and emissions factors in mangroves, tidal salt marshes, and seagrasses. Conservation International, Intergovernmental Oceanographic Commission of UNESCO, International Union for Conservation of Nature. Arlington, Virginia, USA (eds.) (2014).

[b26] Caçador I. . Development of an Angiosperm Quality Assessment Index (AQuA-Index) for ecological quality evaluation of Portuguese water bodies - A multi-metric approach. Ecol Indic 25, 141–148 (2013).

[b27] Braun-Blanquet J. Fitosociologia. In Blum H. (Ed.) Bases para el estudio de las comunidades vegetales Madrid (1979).

[b28] AMBIECO/PLRA. Estudo da Caracterização da Qualidade Ecológica da Ria de Aveiro (Characterization of the Ecological Quality of Ria de Aveiro), Ria de Aveiro POLIS LITORAL, Rehabilitation and Enhancement of the Coastal Zone, AMBIECO (2011).

[b29] Oksanen J. . Vegan: Community ecology package. R package version 1, 15–2 (2009).

[b30] Koop-Jakobsen K. & Wenzhöfer F. The Dynamics of Plant-Mediated Sediment Oxygenation in Spartina anglica Rhizospheres - a Planar Optode Study. Estuar Coast 38, 951–963 (2015).

[b31] Lillebo A. I., Flindt M. R., Pardal M. A. & Marques J. C. The effect of Zostera noltii, Spartina maritima and Scirpus maritimus on sediment pore-water profiles in a temperate intertidal estuary. Hydrobiologia 555(1), 175–183 (2006).

[b32] Sousa A. I., Lillebø A. I., Caçador I. & Pardal M. A. Contribution of Spartina maritima to the reduction of eutrophication in estuarine systems. Environ Pollut 156, 628–635 (2008). 18684544 10.1016/j.envpol.2008.06.022

[b33] Mitsch W. J. & Gosselink J. G. The value of wetlands: importance of scale and landscape setting. Ecological economics. The Values of Wetlands: Landscapes and Institutional Perspectives 35(1), 25–33 (2000).

[b34] Castillo J. M., Leira-Doce P., Rubio-Casal A. E. & Figueroa E. Spatial and temporal variations in aboveground and belowground biomass of Spartina maritima (small cordgrass) in created and natural marshes. Estuarine, Coastal and Shelf Science 78(4), 819–826 (2008).

[b35] Valiela I. . In Concepts and Controversies in Tidal Marsh Ecology (eds Weinstein M. P. & Kreeger D. A.) Ch., 23–36 (Kluwer Academic Publishers, Netherlands, 2000).

[b36] Castillo J. M. . Environmental determination of shoot height in populations of the cordgrass Spartina maritima. Estuar 28(5), 761–766 (2005).

[b37] Castillo J. M., Rubio C., Emilio A., Clemente F. & Figueroa M. E. In Biomass (eds Momba M. & Faizal B.) 1–26 (Sciyo, Croatia, 2010).

[b38] Sousa A. I., Lillebø A. I., Pardal M. A. & Caçador I. The influence of Spartina maritima on carbon retention capacity in salt marshes from warm-temperate estuaries. Mar Poll Bull 61, 215–223 (2010). 10.1016/j.marpolbul.2010.02.018 20304438

[b39] Curado G., Rubio-Casal A. E., Figueroa E., Grewell B. J. & Castillo J. M. Native plant restoration combats environmental change: development of carbon and nitrogen sequestration capacity using small cordgrass in European salt marshes. Environ Monit Assess 185, 8439–8449 (2013). 23591677 10.1007/s10661-013-3185-4

[b40] Hensel P. F., Day J. W. Jr. & Pont D. Wetland Vertical Accretion and Soil Elevation Change in the Rhone River Delta, France. The Importance of Riverine Flooding. J Coastal Res 15(3), 668- 681 (1999).

[b41] Fitzgerald D. M., Fenster M. S., Argow B. A. & Buynevich I. V. Coastal Impacts Due to Sea-Level Rise. Annu. Rev. Earth Planet. Sci. 36, 601–47 (2008).

[b42] Gonzalez-Alcaraz M. N., Egea C., Jimenez-Carceles F. J., Parraga I., Maria-Cervantes A., Delgado M. J. & Alvarez Rogel J. Storage of organic carbon, nitrogen and phosphorus in the soil-plant system of Phragmites australis stands from a eutrophicated Mediterranean salt marsh. Geoderma 185, 61–72 (2012).

[b43] Kirwan M. L. & Blum L. K. Enhanced decomposition offsets enhanced productivity and soil carbon accumulation in coastal wetlands responding to climate change. Biogeosciences 8, 987–993 (2011).

[b44] Kirwan M. L. & Mudd S. M. Response of salt-marsh carbon accumulation to climate change. Nature 489, 550–553 (2012) 23018965 10.1038/nature11440

[b45] Kirwan M. L., Guntenspergen G. R. & Langley J. A. Temperature sensitivity of organic-matter decay in tidal marshes. Biogeosciences 11, 4801–4808 (2014).

[b46] Duarte B. . Modelling sea level rise (SLR) impacts on salt marsh detrital outwelling C and N exports from an estuarine coastal lagoon to the ocean (Ria de Aveiro, Portugal). Ecol Model 289, 36–44 (2014).

[b47] Lopes C. B. . Inputs of organic carbon from Ria de Aveiro coastal lagoon to the Atlantic Ocean. Estuar Coast Shelf Sci 79(4), 751–757 (2008).

[b48] Craft C. . Forecasting the effects of accelerated sea‐level rise on tidal marsh ecosystem services. Front. Ecol. Environ. 7(2), 73–78 (2009).

[b49] Butzeck C. . Sediment Deposition and Accretion Rates in Tidal Marshes Are Highly Variable Along Estuarine Salinity and Flooding Gradients. Estuar Coast 38, 434–450 (2015).

[b50] Lopes C. L. & Dias J. M. Assessment of flood hazard during extreme sea levels in a tidally dominated lagoon. Nat Hazards 77(2), 1345–1364 (2015).

[b51] Lopes C. & Dias J. M. Tidal dynamics in a changing lagoon: Flooding or not flooding the marginal regions. Estuar Coast Shelf S 167, 14–24 (2015).

[b52] Schile L. M. . Modeling Tidal Marsh Distribution with Sea-Level Rise: Evaluating the Role of Vegetation, Sediment, and Upland Habitat in Marsh Resiliency. PLoS One 9(2), e88760. doi: 10.1371/journal.pone.0088760 (2014). 24551156 PMC3923833

[b53] Cui L., Ge Z., Yuan L. & Zhang L. Vulnerability assessment of the coastal wetlands in the Yangtze Estuary, China to sea-level rise. Estuar Coast Shelf S 156, 42–51 (2015).

[b54] Kirwan M. L., Temmerman S., Skeehan E. E., Guntenspergen G. R. & Fagherazzi S. Overestimation of marsh vulnerability to sea level rise. Nature Clim Change 6, 253–260 (2016).

