# Supplementary material for: ‘Blue Carbon’ and Nutrient Stocks of Salt Marshes at a Temperate Coastal Lagoon (Ria de Aveiro, Portugal)
Source: Sci Rep. 2017 Jan 25;7:41225. doi: 10.1038/srep41225 (PMC5264181; doi:10.1038/srep41225)
Supplement: Supplementary Information [file srep41225-s1.pdf]

‘Blue Carbon’ and Nutrient Stocks of Salt Marshes at a Temperate Coastal Lagoon (Ria de Aveiro, Portugal)

Ana I. Sousa[1*], Danielle B. Santos[1,2], Eduardo Ferreira da Silva[2], Lisa P. Sousa[3], Daniel F.R. Cleary[1], Amadeu M.V.M. Soares[1] & Ana I. Lillebø[1*]

[1] Department of Biology & CESAM – Centre for Environmental and Marine Studies, University of Aveiro, Campus Universitário de Santiago, 3810-193 Aveiro, Portugal

[2] Department of Geosciences & GeoBioTec Research Centre, University of Aveiro, 3810-193 Aveiro, Campus Universitário de Santiago, 3810-193 Aveiro, Portugal

[3] Department of Environment and Planning & CESAM - Centre for Environmental and Marine Studies, University of Aveiro, Campus Universitário de Santiago, 3810-193 Aveiro, Portugal

* Corresponding authors: anaisousa@ua.pt (A.I.Sousa); lillebo@ua.pt (A.I.Lillebø)

Supplementary Table S1

Plant species		Site	Month	OM (%)	pH	Temperature (ºC)	Particle size (mean, %)			
							<63 µm	<125 µm	<250 µm	>250 µm < 1000 µm
Juncus maritimus		1	Feb	18.7±4.2	6.36±0.1	13.3±0.3	60.9	16.1	22.9	0.1
			Apr	19.9±8.1	6.03±0.1	13.3±0.1	50.1	27.3	22.3	0.4
			Jun	17.3±1.5	6.33±0.5	22.0±0.0	61.1	18.1	20.6	0.2
			Aug	15.2±3.0	6.51±0.0	21.9±0.1	54.1	28.1	17.8	0.0
			Oct	14.7±2.2	6.69±0.2	16.1±1.0	58.5	14.2	27.1	0.2
			Dec	16.0±2.8	6.95±0.2	7.5±0.4	56.9	20.8	22.1	0.0
		2	Feb	28.2±2.4	5.95±0.5	11.9±0.0	42.5	27.0	29.3	1.2
			Apr	21.5±2.7	5.86±0.0	12.4±0.1	55.9	22.1	21.9	0.2
			Jun	22.4±±1.4	6.20±0.3	18.4±0.1	55.5	19.5	23.8	1.2
			Aug	21.7±4.4	6.14±0.2	20.1±0.1	49.6	23.9	25.7	0.8
			Oct	17.8±0.7	6.90±0.1	14.4±0.6	54.7	19.5	25.5	0.3
			Dec	20.4±1.3	6.79±0.3	8.7±1.5	51.6	22.4	25.2	0.0
		3	Feb	19.3±4.9	6.25±0.2	13.1±0.1	49.8	23.8	25.9	0.4
			Apr	21.2±4.5	6.24±0.1	12.3±0.0	49.3	28.2	21.7	0.8
			Jun	21.0±3.9	6.57±0.2	25.4±0.0	43.1	26.2	30.1	0.6
			Aug	11.9±1.2	6.19±0.2	21.2±0.1	61.9	16.4	21.5	0.2
			Oct	15.8±1.1	6.49±0.2	11.7±0.7	54.4	16.2	29.1	0.3
			Dec	17.5±1.0	6.51±0.3	7.2±0.6	51.7	22.2	25.7	0.0
Spartina maritima		2	Feb	7.5±0.6	6.79±0.1	12.2±0.0	37.8	34.3	27.9	0.0
			Apr	5.9±1.0	7.18±0.0	12.7±0.2	27.2	32.7	40.0	0.1
			Jun	9.8±4.9	7.07±0.2	19.9±0.1	47.4	38.7	13.9	0.0
			Aug	6.8±2.0	7.08±0.1	18.2±0.0	31.0	42.9	26.1	0.0
			Oct	6.0±3.4	6.90±0.1	15.0±0.1	27.1	30.0	42.9	0.0
			Dec	6.0±1.6	6.89±0.1	11.3±0.4	35.6	35.2	29.2	0.0
		3	Feb	12.3±1.6	6.56±0.5	12.0±0.1	53.6	22.3	24.0	0.1
			Apr	10.0±1.1	6.08±0.1	14.3±0.0	64.7	18.2	16.9	0.2
			Jun	11.6±3.1	6.35±0.2	25.4±0.0	54.6	22.5	22.7	0.2
			Aug	8.7±0.3	6.51±0.3	22.6±0.1	62.9	18.9	18.2	0.0
			Oct	9.0±1.0	6.13±0.1	12.3±0.8	62.5	16.8	20.6	0.1
			Dec	10.3±1.4	6.62±0.4	7.9±0.7	61.2	19.8	18.8	0.0

Supplementary Table S1 – Sediment characterization of *Juncus maritimus* and *Spartina maritima* sampled sites along one year (mean ± SD, N=3). Organic matter (OM) is expressed as loss on ignition (LOI).

Supplementary Table S2

Stock compartment		Low marsh	Mid & high marsh			
		Spartina maritima	*Juncus maritimus*	*Halimione portulacoides*[(a)]	*Sarcocornia perennis*[(a)]	*Bolboschoenus maritimus*[(a)]
**C**	Plant	373.4±29.5	1384.1±24.8	843.0	863.1	1141.2
	Sediment	119.5±5.3	132.7±5.8	157.3	152.0	182.9
	**TOTAL**	**492.9±19.8**	**1516.8±26.1**	**1000.3**	**1015.2**	**1324.0**
**N**	Plant	13.4±1.1	47.9±1.2	43.6	34.7	36.2
	Sediment	9.9±1.3	10.8±0.6	38.5	37.4	17.5
	**TOTAL**	**23.3±0.1**	**58.7±1.0**	**109.0**	**98.4**	**66.0**
**P**	Plant	2.2±0.1	8.6±0.1	22.4	14.5	6.0
	Sediment	2.8±0.2	2.8±0.3	**6.9**	**5.4**	4.5
	**TOTAL**	**5.0±0.1**	**11.5±0.3**	**29.3**	**19.9**	**10.5**

[(a)]Estimated from Sousa et al. (2010) data and considering the mean sedimentation rates estimated for Ria de Aveiro (see methods section).

Supplementary Table S2 – Blue C, N and P annual stocks (mean) at the Ria de Aveiro salt marshes, in the plant (above- and belowground material – annual mean (N=18) – g m^{-2}), in the sediment (annual mean (N=18) – g m^{-2} y^{-1}), and the total (plant and sediment – g m^{-2} y^{-1}). Mean values (N=18) and standard error (SE) is also included for *S. maritima* and *J. maritimus* marshes.

Supplementary Figure S1

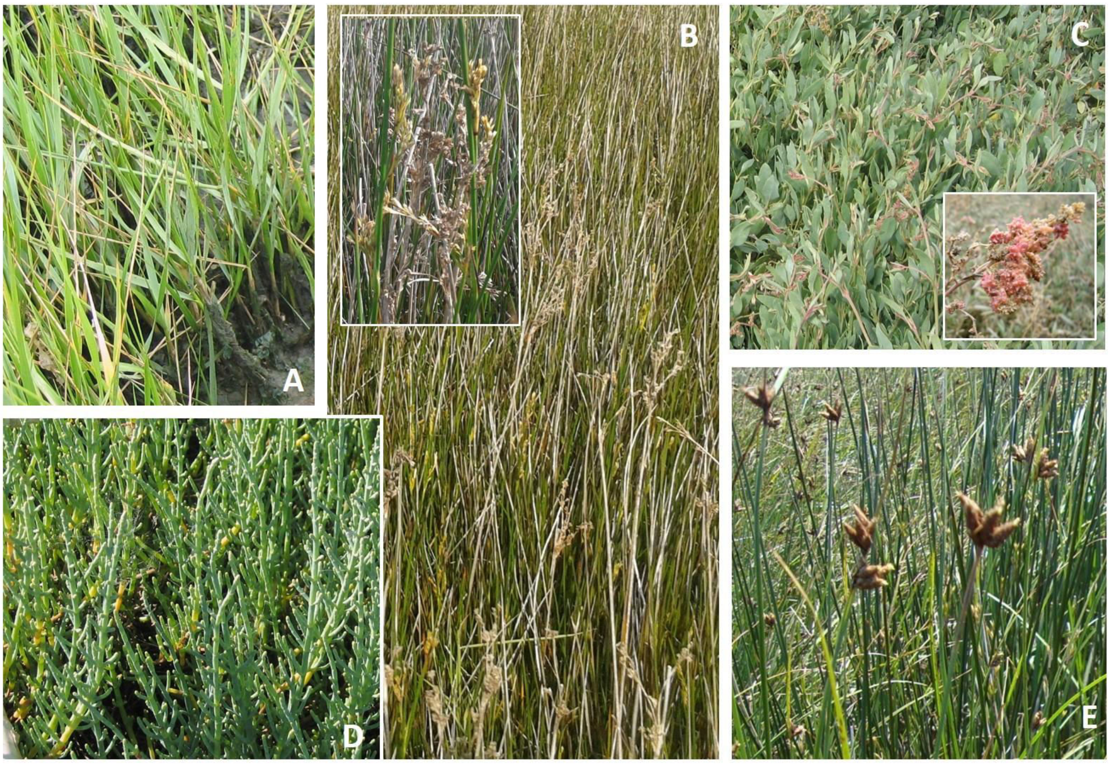

Supplementary Figure S1 – Salt marsh plants: *Spartina maritima* (A), *Juncus maritimus* with flowering in detail (B), *Halimione portulacoides* with flowering in detail (C), *Sarcocornia perennis* (D), *Bolboeschenus maritimus* (E). Pictures copyright: ©A.I. Sousa and ©A.I. Lillebø.

Supplementary Figure S2

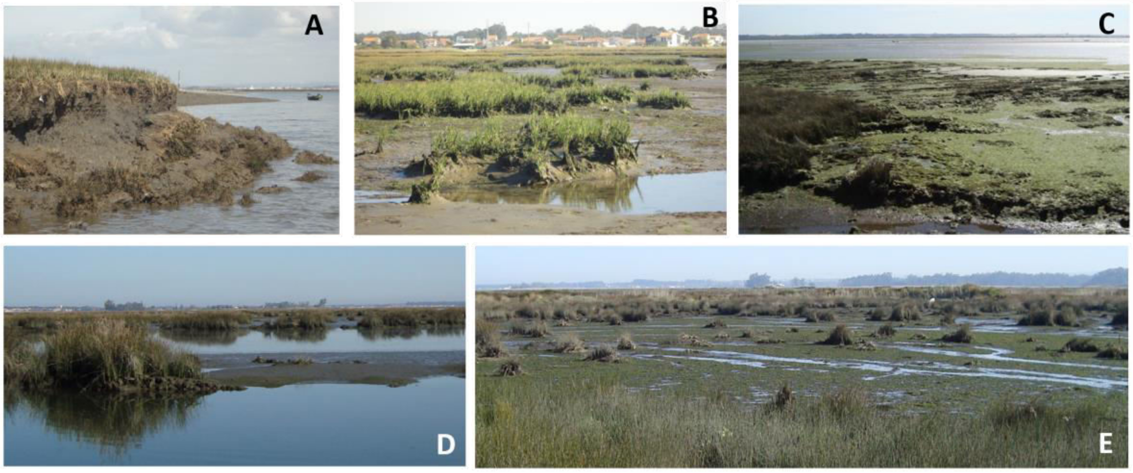

Supplementary Figure S2 – Salt marshes showing evidence of erosion of the shorelines in the Ria de Aveiro. *Spartina maritima* marsh (A, B), eroded shoreline in the marsh (C), and *Juncus maritimus* marsh (D, E). Pictures copyright: ©A.I. Sousa and ©A.I. Lillebø.

Supplementary Figure S3

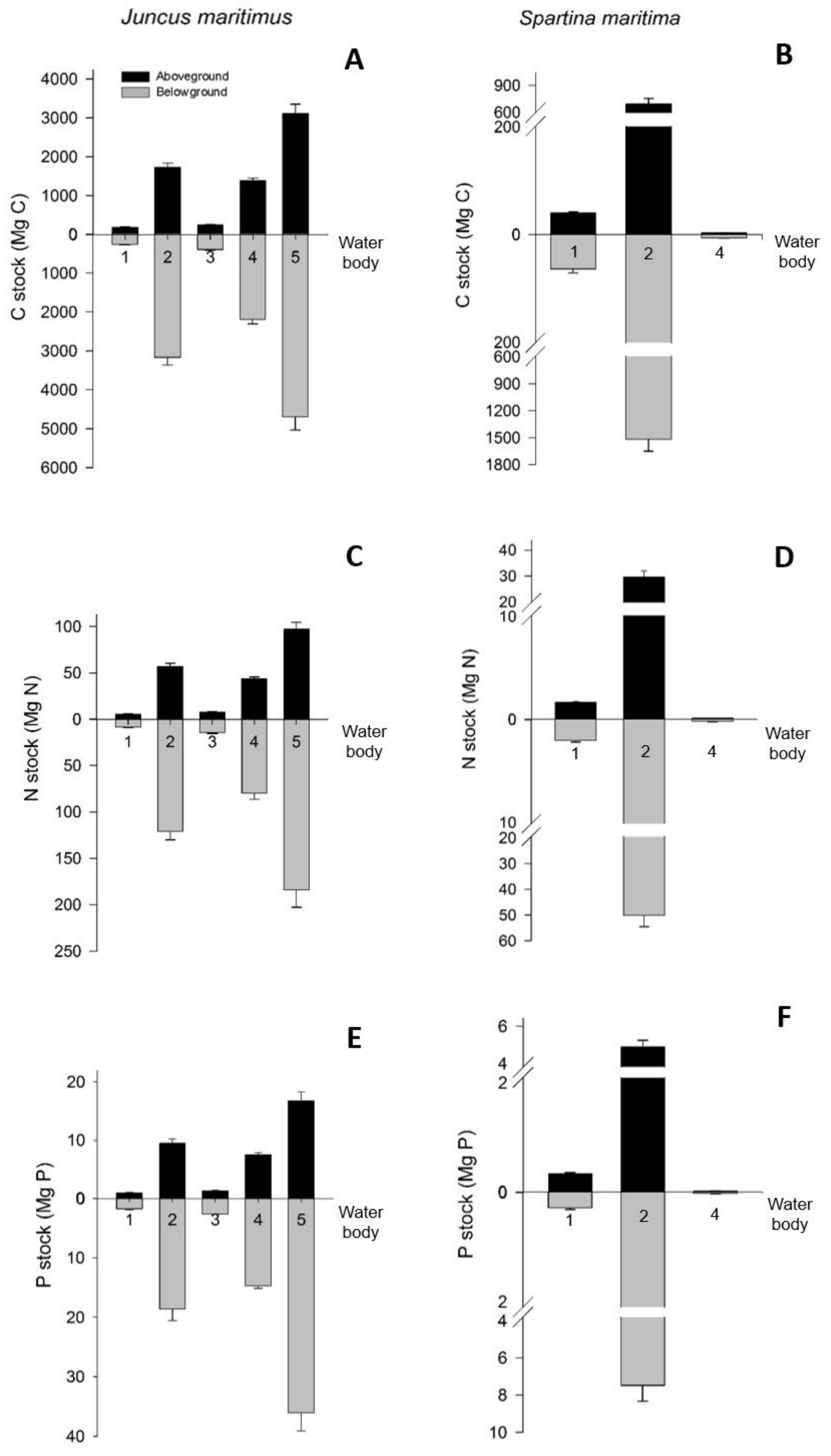

Supplementary Figure S3 – Total C, N and P stocks (mean ± SE, N=18) at the aboveground biomass (leaves and stems) and belowground biomass (roots and rhizomes) in *Juncus maritimus* (A, C, E) and *Spartina maritima* (B, D, F) marshes at Ria de Aveiro, per water body (WB).
